# Pushing the Boundaries of Scientific Research with the use of Artificial Intelligence tools: Navigating Risks and Unleashing Possibilities

**DOI:** 10.3126/nje.v13i1.53721

**Published:** 2023-03-31

**Authors:** Naushad Ahmad Khan, Kudaibergen Osmonaliev, Mohammad Zahed Sarwar

**Affiliations:** 1 Clinical Research, Trauma and Vascular Surgery, Department of Surgery, Hamad General Hospital, Doha, Qatar; 2 Faculty of Medical Sciences, Ala-Too International University, Bishkek, Kyrgyz Republic

**Keywords:** Online Artificial Intelligence, ChatGTP, Scientific research

## Abstract

The language of science is communicated through various modes, such as lectures, informal discussions, conferences, and peer-reviewed publications. Artificial Intelligence (AI) based writing tools, like ChatGPT, have recently become increasingly popular due to natural language processing technology advancements. ChatGPT is an AI language model that can generate text close to human writing, making it suitable for tasks such as summarizing literature, composing essays, and producing statistical studies. This technology has the potential to transform scientific communication, but concerns have been raised about its impact on the integrity of research and the role of human researchers. While this technology has advantages such as accelerating the innovation process and enhancing diversity in scientific viewpoints, it is important for the scientific community to debate and envision the consequences of its use. Publishers are working to develop guidelines for its application, which may be capable of future activities such as experiment design and peer review. As we enter the early stages of the AI revolution, it is imperative that the scientific community engages in discourse and contemplate the potential outcomes of this potentially transformative technology. With this in mind, we have outlined relevant topics as a starting point for discussion.

## Background

The year 2022 saw a return to pre-pandemic normalcy, with increased travel and in-person activities, including scientific research and conferences. As the year ends, it is important to review the significant advancements in science and technology that have impacted scholarly writing and publishing. Artificial Intelligence (AI) based writing tools like ChatGPT have recently become increasingly popular due to advancements in natural language processing. Artificial Intelligence (AI) based writing tools like ChatGPT have recently become increasingly popular due to advancements in natural language processing. As AI technologies continue to develop and become more publicly available, they are projected to become increasingly incorporated into scientific writing and publishing.

Since the release of ChatGPT (Chat generative Pre-trained Transformer; Open AI, San Francisco, CA, USA), an AI-powered chatbot introduced in November 2022 [1], has made a considerable influence in the academic world and it has become clear that this technology will significantly impact the way researchers conduct their work. ChatGPT is an excessive profanity model trained to utilize a large quantity of online text data. Pattern recognition is the basis of GPT, an AI that can learn from online content to provide answers to user inquiries in response to a written prompt. This model can generate text that is close to human writing, making it suitable for tasks like language translation, text summarization, and question answering. Notably, ChatGPT is being used by researchers for a variety of applications, including composing essays, summarizing literature, improving papers, detecting research gaps, and producing computer code and statistical studies [2] It has also been used in academic contexts to generate research papers and graphic features such as figures and tables and is more often seen in such publications [3] . A recent report published in Nature magazine revealed that some researchers have begun employing chatbots to aid them with activities, including organizing their ideas, giving comments on their work, developing code, and summarizing research material [4] .

This technology may have a substantial influence on science and society. Since its inception, the ChatGPT has been mentioned in multiple preprints and published articles with authorship credits. This has spawned a discussion and debate over the function of AI tools in published literature and whether they need to be recognized as authors among journal editors, academics, and publishers. Publishers are working to develop guidelines for the application [5]. With the advancement of this technology, it is plausible that it would be capable of activities like experiment design, paper composition and finalization, peer review, and helping in editorial judgments on manuscript acceptance or rejection [6] .

This form of conversational AI integration in research and publication has the potential to change the discipline, presenting both advantages and drawbacks. It can accelerate the innovation process, shorten the time it takes to publish, and enhance equality and diversity in scientific viewpoints by assisting people who struggle with writing [7]. However, there are concerns that it would reduce the integrity of the research and transparency, as well as fundamentally alter the role of human researchers. As we embrace the early phases of the AI revolution, it is critical that the scientific community debate and envision the consequences of this potentially revolutionary technology that awaits us. In this context, we summarize the pertinent topics and provide a starting point for the discussion.

### Benefits to embrace: Improved Efficiency and productivity

Conversational AI is becoming increasingly important as demand and competitiveness in academic sectors increase. Chatbots provide an advantage by allowing tasks to be performed rapidly, whether for PhD students working on their dissertations, researchers in need of a quick literature review for a funding request, or peer-reviewers who are short on time.

If artificial intelligence chatbots can help with these activities, academics will have more time to focus on new research and projects. This might have a huge influence on innovation, leading to important advances in a wide range of fields. Despite present bias, dependability, and accuracy limitations, we feel this technology has immense promise. It is critical to assess and improve the validity of language models so that researchers may utilize the technology in an effective and ethical manner.

The incorporation of AI technology may result in a shift in the academic skill set. On the one hand, AI can improve academic learning by offering feedback to improve student writing and reasoning abilities. However, technology may also reduce the need for some abilities, such as manual literature search. Furthermore, it may bring new abilities such as developing and producing prompts for conversational AI models. The loss of some expertise may not be an issue (since most researchers no longer undertake statistical analysis manually), but it is critical for the academic community to carefully analyze which skills and attributes are essential for researchers to possess.

If we only emphasize performance and efficiency, the role and impact of individuals may decline as AI technology advances. In the future, AI chatbots might generate ideas, devise methodologies, perform tests, analyze and interpret data, and even write articles. They might also be used to evaluate and assess publications in place of human editors and reviewers. Although we are still a long way from this reality, it is clear that conversational AI technology will have an increasing influence on all parts of scientific publication.

As a result, it is critical for researchers, particularly ethicists, to debate the trade-off between using AI to accelerate knowledge creation and the possible loss of human capability and control in the research process. Creativity, originality, education, training, and human relationships will almost certainly remain important in performing relevant and novel research.

### The Consequences of Unbridled Creativity and artificial authorship: The Need for Ethical Consideration in Science

Although Conversational AI-supported language models have evolved over time, recent improvements in the amount and quality of data sets, along with advanced techniques for calibrating with human feedback, have suddenly made these models substantially more effective. Currently, the impact of Conversational AI on medical publications is unknown. However, many researchers believe that the application of Conversational AI may generate serious ethical concerns. The technology has been demonstrated by researchers to be capable of successfully passing medical license examinations, but this has been surrounded by a number of ethical issues.

In a preprint on utilizing the technology for medical education that was published on the medical repository medRxiv in December of last year, ChatGPT is listed as one of 12 authors [8] . This application was listed as a co-author on an editorial that appeared in the journal Nurse Education in Practice [9] . An editorial article listed ChatGPT as a co-author in the journal Oncoscience in research published from Hong Kong[10] . In June 2022, a French preprint server named HAL published a fourth essay co-written by an earlier chatbot by the name of GPT-3 and was later accepted for publishing in a peer-reviewed journal [11] .

The utilization of conversational AI for specialized research may bring inaccuracies, copyright concerns, attribution, plagiarism, and authorship. When paired with sophisticated AI language models, the present algorithms for creating phony research papers and documents might become much more effective. The output of fraudulent research articles might be significantly increased by a text-generating system that is quick to deploy and uses well-structured language, making it even more difficult to spot them. The publishing sector has already been impacted by this problem and using AI might make things considerably worse.

These concerns are especially pressing because it is currently difficult for human readers, and anti-plagiarism software to discern between AI-generated and human-written content. ChatGPT has already been designated as an author in several academic domains, bringing into focus the ever-increasing urgency of scientific publishing institutions to develop and apply rigorous AI author standards. Moreover, whether generative AI fits the International Committee of Medical Journal Editors' criterion for authorship is still up for contention. Can a chatbot actually accept responsibility for its job and grant content approval? The Committee on Publication Ethics developed standards for editorial decision-making using AI. At the same time, the International Association of Scientific, Technical, and Medical Publishers, which represents academic publishers, released a white paper on AI ethics [12].Moreover, the World Association of Medical Editors also published its recommendations for the use of chatbots like ChatGPT in academic articles last month, and it recommended that Journal editors should have access to new techniques to identify content that has been created or edited by AI.

Recently, Nature [5], Science [13] and JAMA journals [14] stated that AI-generated text, including that produced by ChatGPT, cannot be used as authorship in papers about language models, machine learning, or related technologies. If authors utilize these tools to aid in writing or manuscript preparation, they must take accountability for the accuracy of the generated content. If AI was utilized in a formal research design or methodology, it should be acknowledged in either the acknowledgment or methods section of the paper, which should include a description of the edited or generated content and details of the language model or tool used, including its name, version, extension number, and manufacturer.

There are concerns regarding the originality of research produced by AI on a broader level. Is an AI-generated scientific text still regarded as original if it was trained on information authored by humans? Who is the owner of the intellectual property for such a work? These issues raise similar worries about who would profit from AI's creative output, which is reflective of the continuing discussion concerning AI-generated art. Will an AI system with access to all of the literature be able to appropriately credit the relevant scientific work for its results, or will using decentralized literature sources cause the emphasis to move away from human authorship and toward a new paradigm?

It is evident that to address these challenges, there needs to be a clear distinction between the acceptable and unacceptable use of AI-generated content. The fact that AI can already produce articles that might be considered suitable for peer review by some authors highlights the pressing need for ethical guidelines in the usage of AI-generated text in scientific literature. It is very necessary to have in-depth conversations regarding the regulations governing authorship as the state of technology continues to advance and become more pervasive. Recent statements by Elsevier and other prominent publishers assert that these technologies should be used exclusively to improve the readability and language of the article and that their use is documented in the publication. Furthermore, the authors must manually evaluate any AI-generated output. It is also stated that AI tools should not be recognized or credited as authors or co-authors since they are incapable of adopting the responsibility and accountability that authorship entails.

### Drawbacks: Fluent but not consistently precise

There is a growing consensus among researchers that conversational AI technologies have the potential to speed processes such as writing papers and grants, but only under human supervision. They believe that scientists will no longer be required to spend time crafting lengthy grant applications and presentations. However, they warn that these chatbots must be more trustworthy when responding to inquiries since they may produce misleading replies. The unreliability is attributable to the construction of these tools. For instance, models like ChatGPT discovers statistical patterns in enormous quantities of online text, which may contain mistakes, biases, and obsolete data.

In the case of writing or revising portions of an academic paper, chatbots are good at merely regurgitating the most stylistically reasonable response based on the facts they have observed [15] . It is crucial to acknowledge that ChatGPT's effectiveness hinges upon its pre-existing database and content. As of the writing of this editorial, its limitation lies in the absence of information published or posted post-2021, thereby curbing its applicability in developing introductions, recent reviews, and perspectives. As a result, these technologies frequently generate mistakes and misleading information, particularly in technical domains where training data is scarce. In addition, these models are unable to specify the source of their data. They may fabricate citations and references when producing academic papers, prompting experts to conclude that they cannot be relied upon to give correct information or credible references.

Therefore, in terms of critiques and viewpoints, ChatGPT falls short as it lacks the analytical prowess that befits a scientist and the experiential insights that shape our understanding. As scientists, our paramount apprehension is that these AI language bots are ill-equipped to assimilate novel information, conjure up revelations, or engage in profound scrutiny, ultimately curtailing the scope of scholarly discourse. Nevertheless, it's important to consider if the existing AI technological constraints are long-lasting or merely passing. Unexpectedly, recent developments in the use of AI in challenging strategic games have demonstrated that it can outperform humans in activities formerly believed to be computationally intractable [16, 17] .

The rise of AI automation might also result in the loss of unique writing styles, replacing them with a uniform style. This raises questions about whether the homogenization of writing is a step towards greater understanding or a loss of distinctive features. The outcome could depend on how AI systems follow the “mean” of their training data, potentially suppressing valuable deviations [14]. Regulating the randomness in AI-generated content may help prevent this homogenization. However, it remains to be seen if these systems can replicate the diversity of perspectives and voices provided by human scientists.

Finally, while considering the long-term effects of AI on scientific publishing, it is critical to examine the potential biases in AI models. While human researchers are not immune to prejudice, having numerous viewpoints helps lessen individual biases' consequences. Furthermore, the ability to trace authorship allows for correcting systemic biases. However, depending on a small number of AI systems for scientific production has the potential to amplify biases and rapidly spread them around the globe, circumventing the process of individual scrutiny that is frequently employed to discover these biases. Given these concerns, it is essential to think about and regulate the transparency of AI models and their training data. Some of these issues may be addressed with minor modifications, while others may require more significant changes or restrictions on the use of these technologies. It is clear that AI is poised to play a major role in scientific publishing and will bring about significant lasting changes.

Therefore, we call for action for the establishment of a worldwide forum to address the development and ethical use of AI tools for research purposes. To kickstart this endeavor, we suggest a confluence of important participants, including experts from diverse scientific areas, IT businesses, significant funding organizations, research academies, publishers, non-governmental groups, and privacy and law experts. This conversation should result in real, practical proposals and instructions for all parties.

### Concluding remarks: Balancing Creativity and Responsibility in Scientific Pursuits

The language serves as the conduit for scientific communication. In the realm of science, the means of linguistic expression are manifold, encompassing diverse modes such as classroom lectures, casual chats between scientists, well-crafted presentations at symposia, and, culminating in the pinnacle of scientific discourse, the rigorous evaluation of peer-reviewed publications. Science has maintained its credibility by being open about its techniques and evidence from its inception. As scientists investigate the cutting-edge field of advanced AI chatbots, publishers need to recognize the practical applications of these tools and establish clear rules to prevent misuse. Scientists should explore how to maintain transparency and confidence in the knowledge-generating process if they or their colleagues utilize software that acts in a secretive and ambiguous manner. As of now, the widespread adoption of ChatGPT is inevitable, but if used recklessly and without proper oversight, it poses a threat to the academic publishing industry. To prevent this, it is necessary to take a more thoughtful and diligent approach to model training and invest in AI output detection systems. While ChatGPT has the potential to revolutionize the field, we still need to prepare to use it effectively and comprehensively.

As text-detection mechanisms evolve to detect ChatGPT-generated content [18], these AI-powered language bots will concurrently progress in both performance and intricacy, making it progressively challenging to monitor their utilization. The impact of AI-powered bots on science cannot be ignored, as it has the potential to stifle creativity and results in lacklustre and mundane research unless used solely as a catalyst for inventive ideas. Although AI tools excel at repeating established knowledge, they fall short in recognizing and producing novel findings. In fact, they may even struggle in determining if a new discovery is genuine, aberrant, or groundbreaking. We must remember that these AI-powered tools are a work in progress and are rapidly advancing, with new advancements appearing every month. Hence, it may become more prevalent in the future of academic writing; nevertheless, this trend should be carefully balanced with human oversight and discretion. Scientific research must maintain transparency in its processes and uphold the principles of integrity and accuracy in its reporting, which is crucial for the progress and advancement of science. Ultimately, the choices made by researchers in utilizing these innovations will shape both their and our future. It is unreasonable to believe that we have seen the end of these advancements in early 2023, as it is only the beginning.
